# High mortality from neo-adjuvant chemo-radiotherapy for rectal cancer needs to be evaluated against the alternative of adjuvant therapy

**DOI:** 10.1038/sj.bjc.6603338

**Published:** 2006-10-03

**Authors:** S Papagrigoriadis

**Affiliations:** 1 Department of Colorectal Surgery, King's College Hospital Denmark Hill London, London SE5 9RS, UK

**Sir,**

The results of the trial of Klautke *et al* (2006). ‘Concurrent chemoradiation with capecitabine and weekly irinotecan as preoperative treatment for rectal cancer: results from a phase I/II study’ create concerns regarding the toxicity of their scheme.

Although they report good results in terms of local disease control, their mortality of two out of 28 patients or 7.1% is worryingly high.

Similar concerns are raised by another recent study on neo-adjuvant radio-chemotherapy on rectal cancer, by Chau *et al* (2006). ‘Neo-adjuvant capecitabine and oxaliplatin followed by synchronous chemoradiation and total mesorectal excision in magnetic resonance imaging-defined poor-risk rectal cancer’. In that study four out of 77 patients or 5.1% died during the neo-adjuvant treatment.

Although neo-adjuvant treatment has often impressive temporary results in local response it should be kept in mind that the so far proven survival benefit is small and around or below 10%. If this has to be counterbalanced with a 5–7% mortality risk from neo-adjuvant treatment plus 3–5% risk of major rectal surgery, then we may end up with the combined risks of the two modalities outweighing the long-term improvement of survival probability.

Adjuvant chemo-radiotherapy carries similar toxicity but it allows more accurate selection of those patients who have pathological, rather than radiological, staging plus other pathological risk factors such as infiltrated margins, perineural or lymphovascular invasion. The risk of toxicity on those patients of proven high risk would be more acceptable to both clinician and patient.
